# Supplementary material for: Molecular mechanism of biased signaling at the kappa opioid receptor
Source: Nat Commun. 2023 Mar 11;14:1338. doi: 10.1038/s41467-023-37041-7 (PMC10008561; doi:10.1038/s41467-023-37041-7)
Supplement: Supplementary file 1 — Supplementary Information [file 41467_2023_37041_MOESM1_ESM.pdf]

Supplemental Materials

Molecular mechanism of biased signaling at the kappa opioid receptor

Amal EI Daibani[1,2], Joseph M. Paggi[3], Kuglae Kim[4,5], Yianni D. Laloudakis[3], Petr Popov[6], Sarah M. Bernhard[1,2], Brian E. Krumm[4], Reid H.J. Olsen[4], Jeffrey Diberto[4], F. Ivy Carroll[7], Vsevolod Katritch[8], Bernhard Wunsch[9], Ron O. Dror[3,10*], Tao Che[1,2*]

[1]Department of Anesthesiology, Washington University School of Medicine, Saint Louis, MO, USA

[2]Center for Clinical Pharmacology, University of Health Sciences & Pharmacy at St. Louis and Washington University School of Medicine, Saint Louis, MO, USA

[3]Department of Computer Science, Stanford University, Stanford, CA, USA

[4]Department of Pharmacology, University of North Carolina School of Medicine, Chapel Hill, NC, USA

[5]Present address: Department of Pharmacy, Yonsei University, Incheon, 21983, Republic of Korea

[6]iMolecule, Skolkovo Institute of Science and Technology, Moscow, Russia

[7]Research Triangle Institute, P.O. Box 12194, Research Triangle Park, NC 27709, USA

[8]Department of Biological Sciences, University of Southern California, Los Angeles, CA, USA

[9]Institut für Pharmazeutische und Medizinische Chemie, Universität Münster, Correnssstraße 48, 48149, Münster, Germany

[10]Departments of Molecular and Cellular Physiology and of Structural Biology, Stanford University School of Medicine, Stanford, CA, USA

Supplementary Information

Supplemental Figure 1. **a**. Size-exclusion chromatography of KOR WT and computation-designed mutants. **b**. Thermostability of KOR mutants compared with the wild type. **c**. The thermostabilized mutation S324$^{7.47}$C does not significantly change the function of nalfurafine. Data are expressed as the mean ± SEM of three independent experiments (n = 3 experiments each done in duplicate). **d**. 2Fo-Fc and Fo-Fc map of nalfurafine in the binding pocket of KOR. **e**. The binding affinity of nalfurafine in the presence of Nb39 or Gi1. **f** and **g**. The effect of D138$^{3.32}$A on the functional activity of nalfurafine in G protein-mediated cAMP inhibition assay and Tango arrestin recruitment assay. Data are expressed as the mean ± SEM of three independent experiments (n = 3 experiments each done in duplicate). Values in each plot were summarized in Supplemental Table 3.

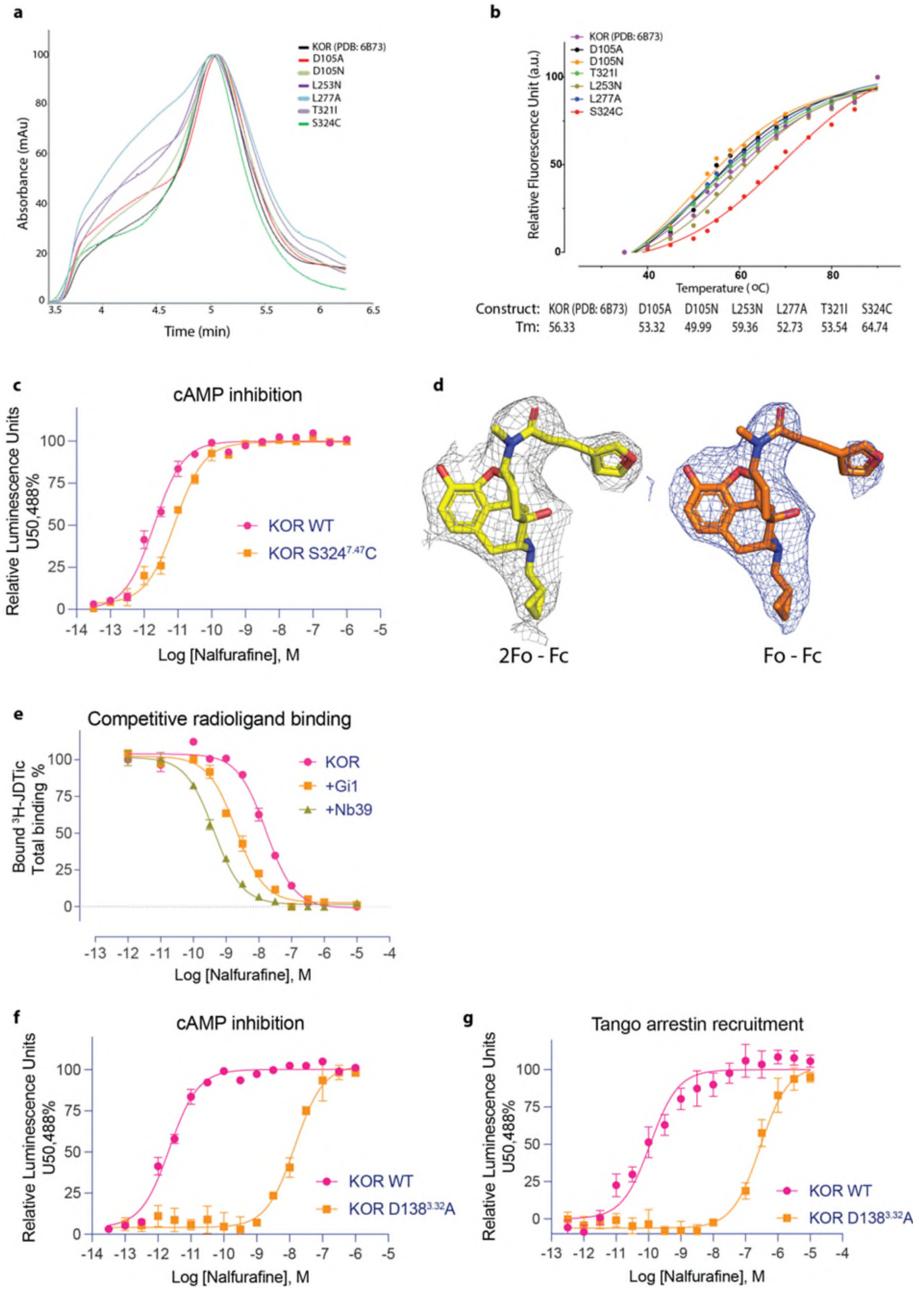

Supplemental Figure 2. **a**. The extracellular loop 2 (EL2) adopts different orientation between nalfurafine and the antagonist JDTic. **b**. The binding pocket of JDTic-bound KOR. **c**. The binding pocket of MP1104-bound KOR. **d**. The binding pocket of nalfurafine-bound KOR. **e**. The EL2 “lid” residues do not significantly affect the functional activity of nalfurafine. **f**. The EL2 “lid” residues accelerate the dissociation of nalfurafine from KOR. **g** and **h**. The effect of G319$^{7.42}$L and Y320$^{7.43}$L on the functional activity of nalfurafine in G protein-mediated cAMP inhibition assay and Tango arrestin recruitment assay. Data are expressed as the mean ± SEM of three independent experiments (n = 3 experiments each done in duplicate). Values in each plot were summarized in Supplemental Table 4.

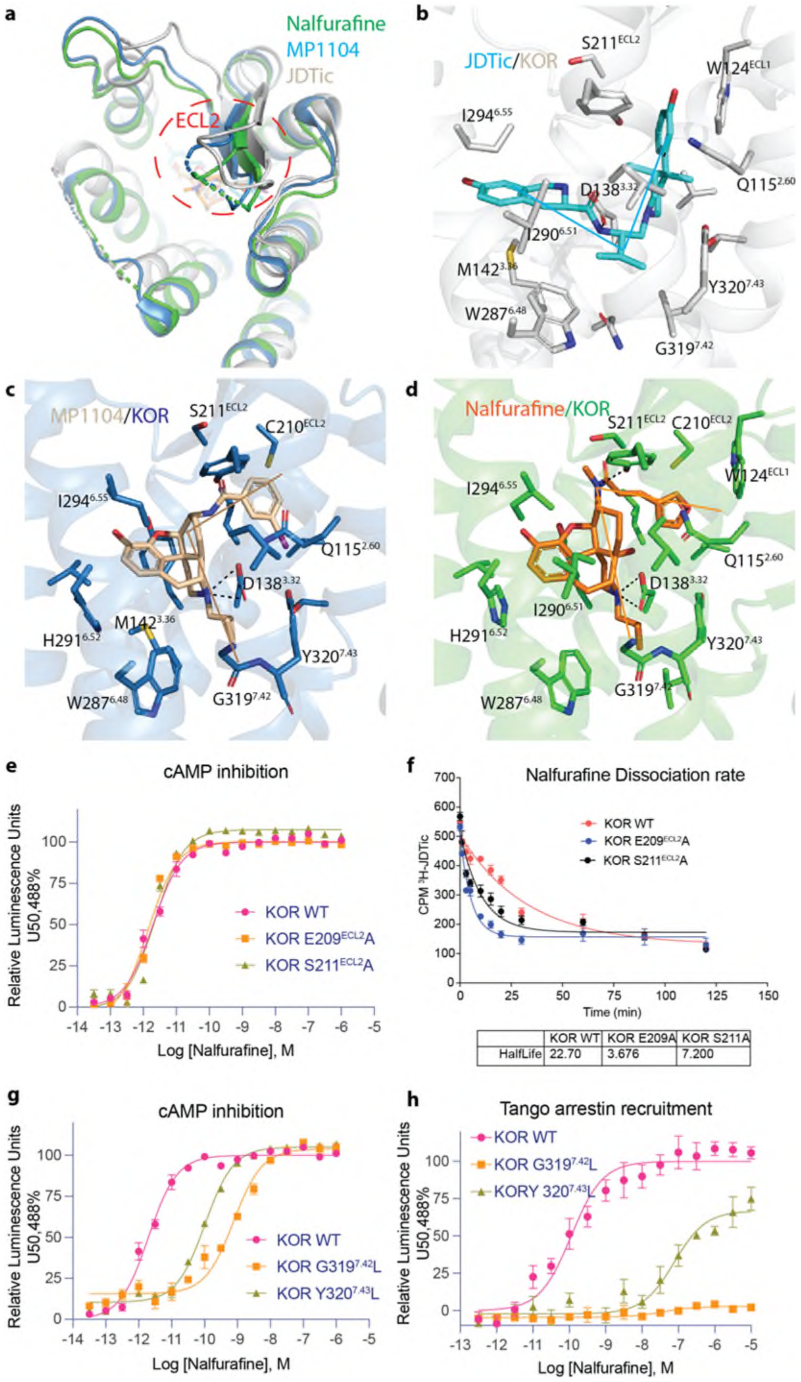

Supplemental Figure 3. **a**. The W287$^{6.48}$ adopts different positions upon the agonist nalfurafine and the antagonist JDTic binding. **b**. The effect of W287$^{6.48}$A on the nalfurafine-mediated G protein activation and arrestin recruitment. Data are expressed as the mean ± SEM of three independent experiments (n = 3 experiments each done in duplicate). **c**. The furan ring of nalfurafine forms hydrophobic interactions with the ‘triad’ residues F114$^{2.59}$/W124^{ECL1}/V134$^{3.28}$. **d**. The effects of mutations in the triad pocket, F114$^{2.59}$L, W124^{ECL1}L or V134$^{3.28}$A, on the functional activity of nalfurafine in G protein-mediated cAMP inhibition assay and Tango arrestin recruitment assay. Data are expressed as the mean ± SEM of three independent experiments (n = 3 experiments each done in duplicate). Values in each plot were summarized in Supplemental Table 5.

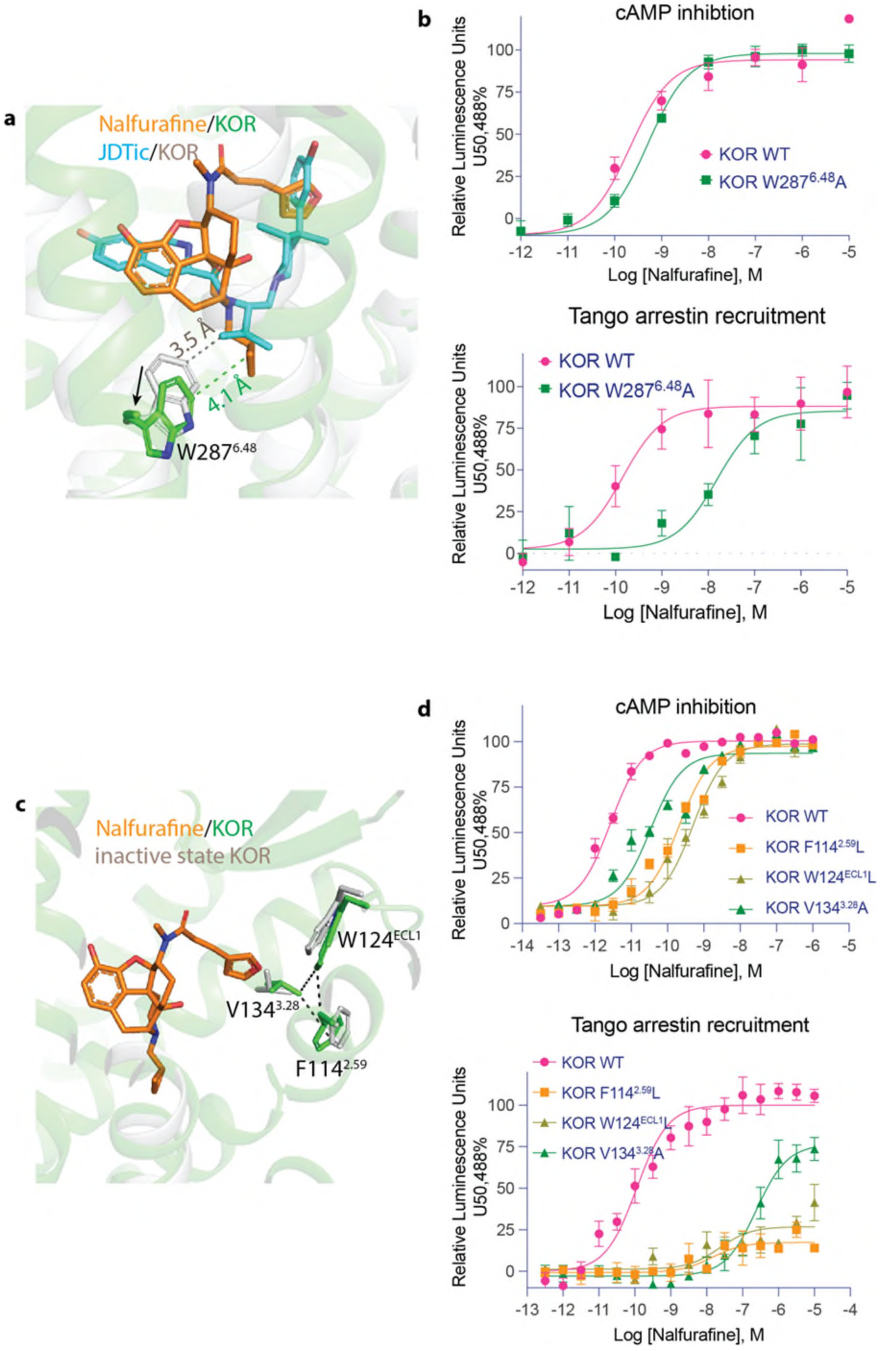

Supplemental Figure 4. a. G protein-biased activity (nalfurafine) and arrestin-biased activity (WMS-X600) confirmed by secondary BRET assays. **b**. Average ligand and binding pocket conformations in MD simulations. In cyan, the simulation frame where the ligand and binding site are most similar (lowest RMSD) to their average coordinates across all MD simulations with the indicated ligand bound. In magenta: the crystallographic pose of nalfurafine and the docked poses for U50,488 and WMS-X600, which were used to initiate the simulations. **c**. Differential effects of binding pocket residue Q115$^{2.60}$ mutation on the functional activity of nalfurafine and WMS-X600. **d**. Differential effects of binding pocket residue K227$^{5.39}$A mutation on the functional activity of nalfurafine and WMS-X600. Data are expressed as the mean ± SEM of three independent experiments (n = 3 experiments each done in duplicate). **e**. Disruption of the K227$^{5.39}$–E297$^{6.58}$ salt bridge results in an increased distance between the extracellular ends of TM5 and TM6 (measured as distance between the CAs of E297$^{6.58}$ and F225$^{5.37}$). Values in each plot were summarized in Supplemental Table 7.

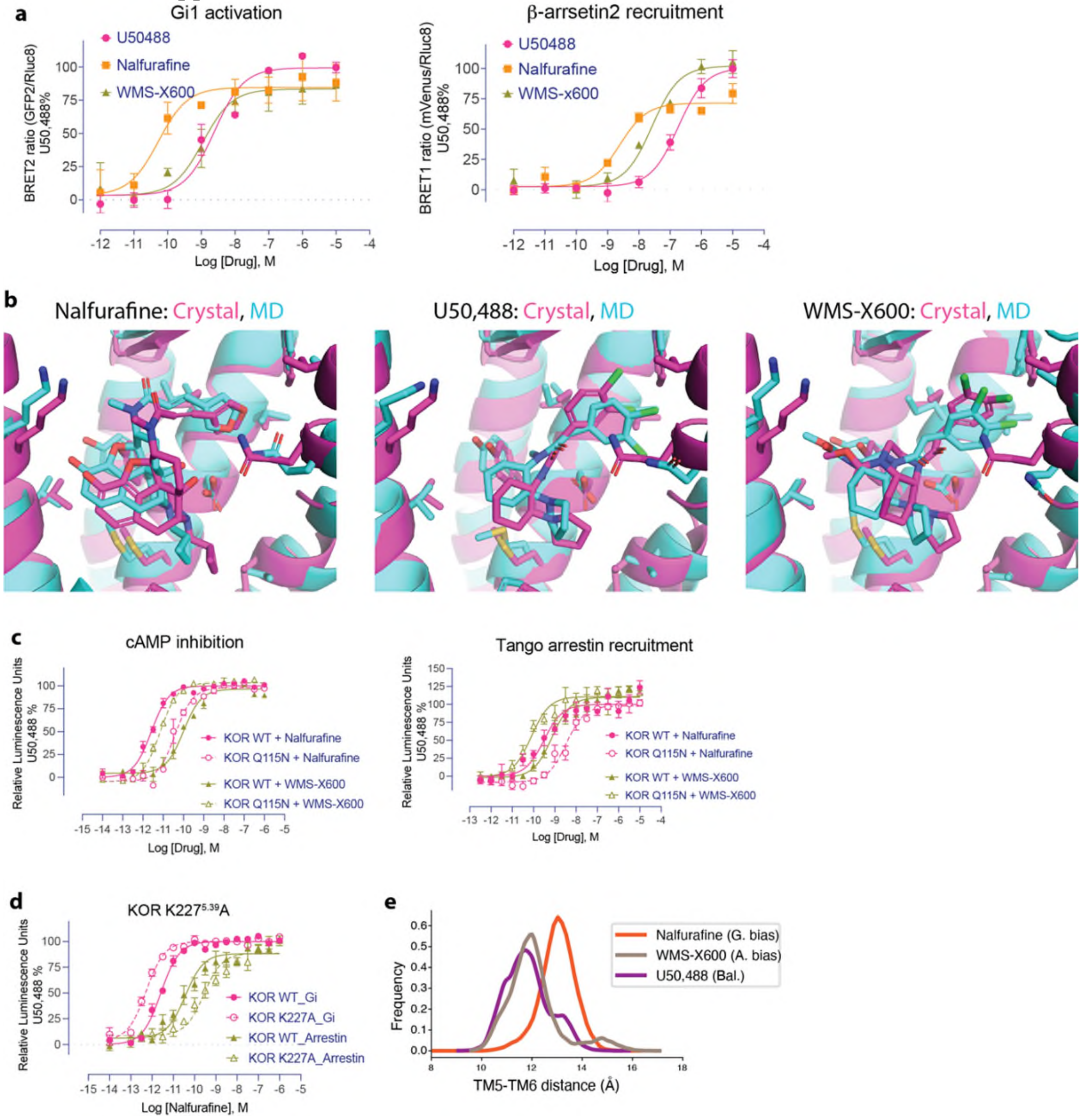

Supplemental Figure 5. Interaction with the 'toggle switch' residue W287$^{6.48}$ displays ligand-specific effects in signaling. **a**. Comparison of the vertical displacement of the CZ atom of W287$^{6.48}$ for the three ligands. **b**. Ligand-specific vertical displacement of W287$^{6.48}$. **c**. The vertical displacement of W287$^{6.48}$ is coupled to the rotation of TM7 through the N322$^{7.45}$ side chain. **d** and **e**. W287$^{6.48}$ poses different effects on WMS-X600 (**d**) and U50,488 (**e**) mediated G protein or arrestin signaling. Data are expressed as the mean ± SEM of three independent experiments (n = 3 experiments each done in duplicate). Values in each plot were summarized in Supplemental Table 8.

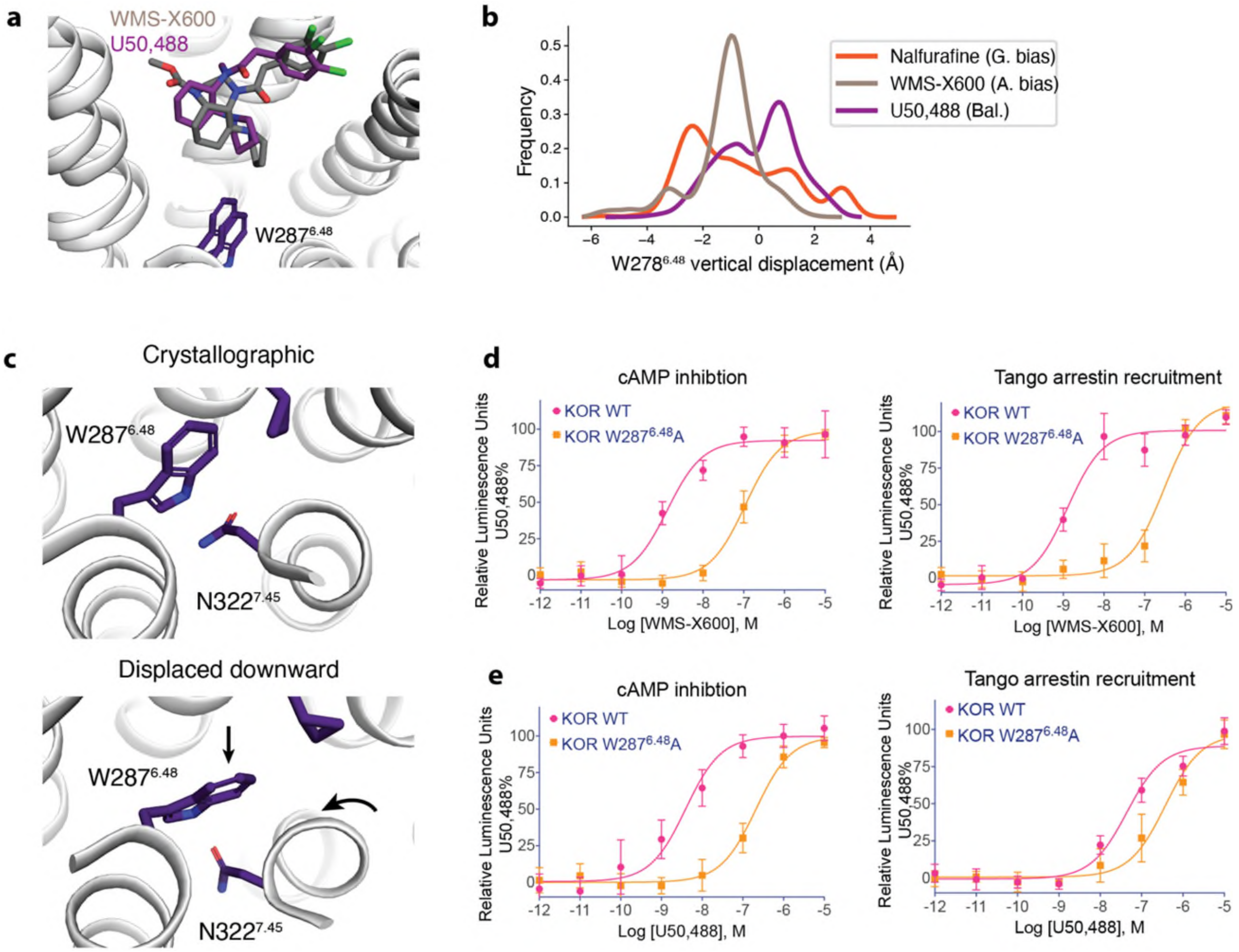

Supplemental Figure 6. a. MD simulation convergence analysis. Each of the left and right columns are analogous to Figure 4. The left column uses only the simulation frames from 1.5 μs to 2.25 μs and the right column uses only simulation frames from 2.25 μs to 3.0 μs. Note that our simulations are 3.0 μs, and we drop the first 1.5 μs to allow the simulations to converge, so this corresponds to the first and second half of the frames shown in Figure 4. The similar populations of each state in either time split suggests that the populations would not change significantly if the simulations were extended. **b**. The G protein-biased KOR agonist, 6'-GNTI, likely has a similar effect on K227$^{5.39}$ and induces a similar conformational state as nalfurafine does.

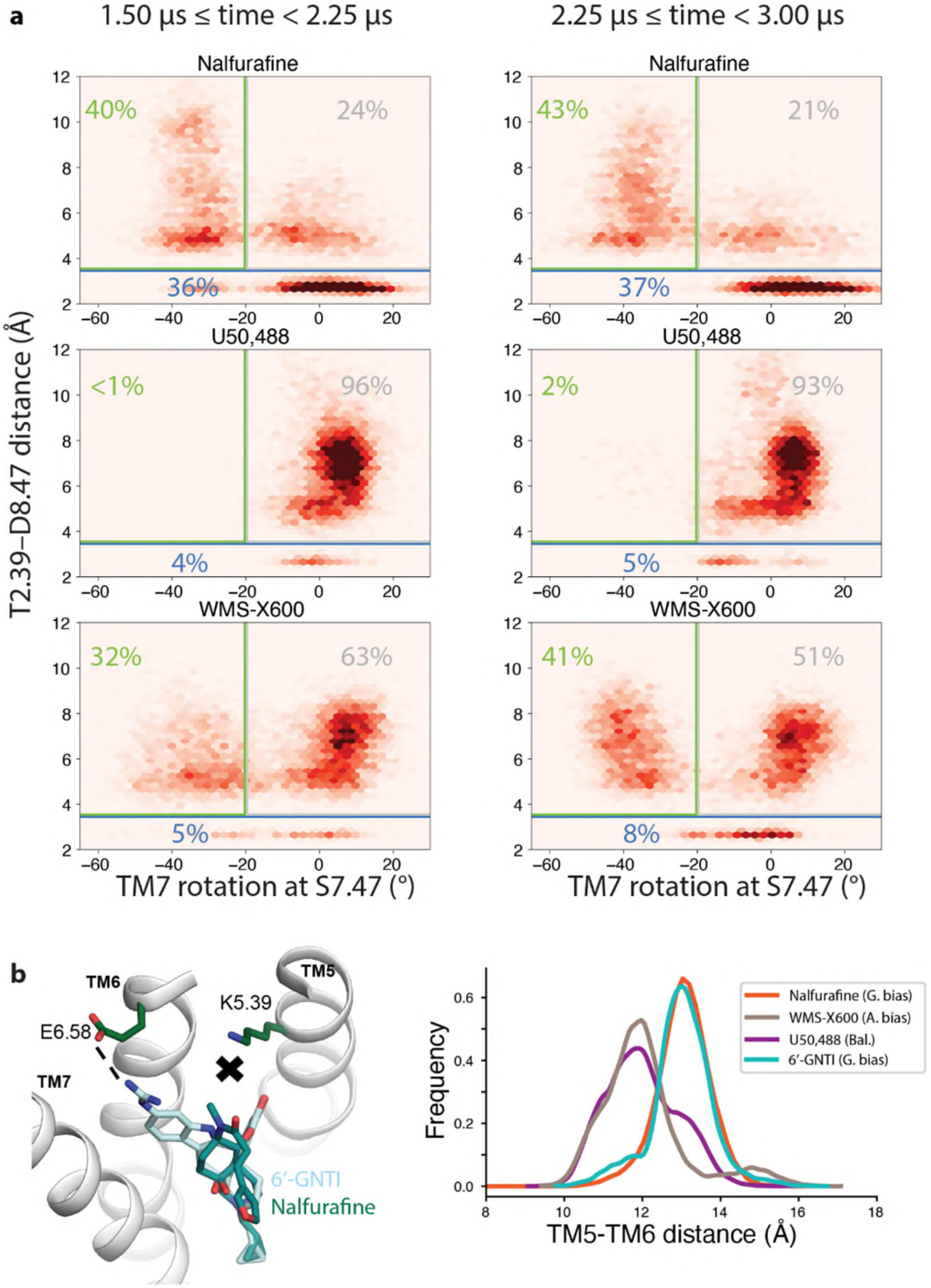

Supplemental Figure 7. Effects of indicated residues on the KOR-G protein subtype interactions. Data are expressed as the mean ± SEM of three independent experiments (n = 3 experiments each done in duplicate). Values in each plot were summarized in Supplemental Table 10-11.

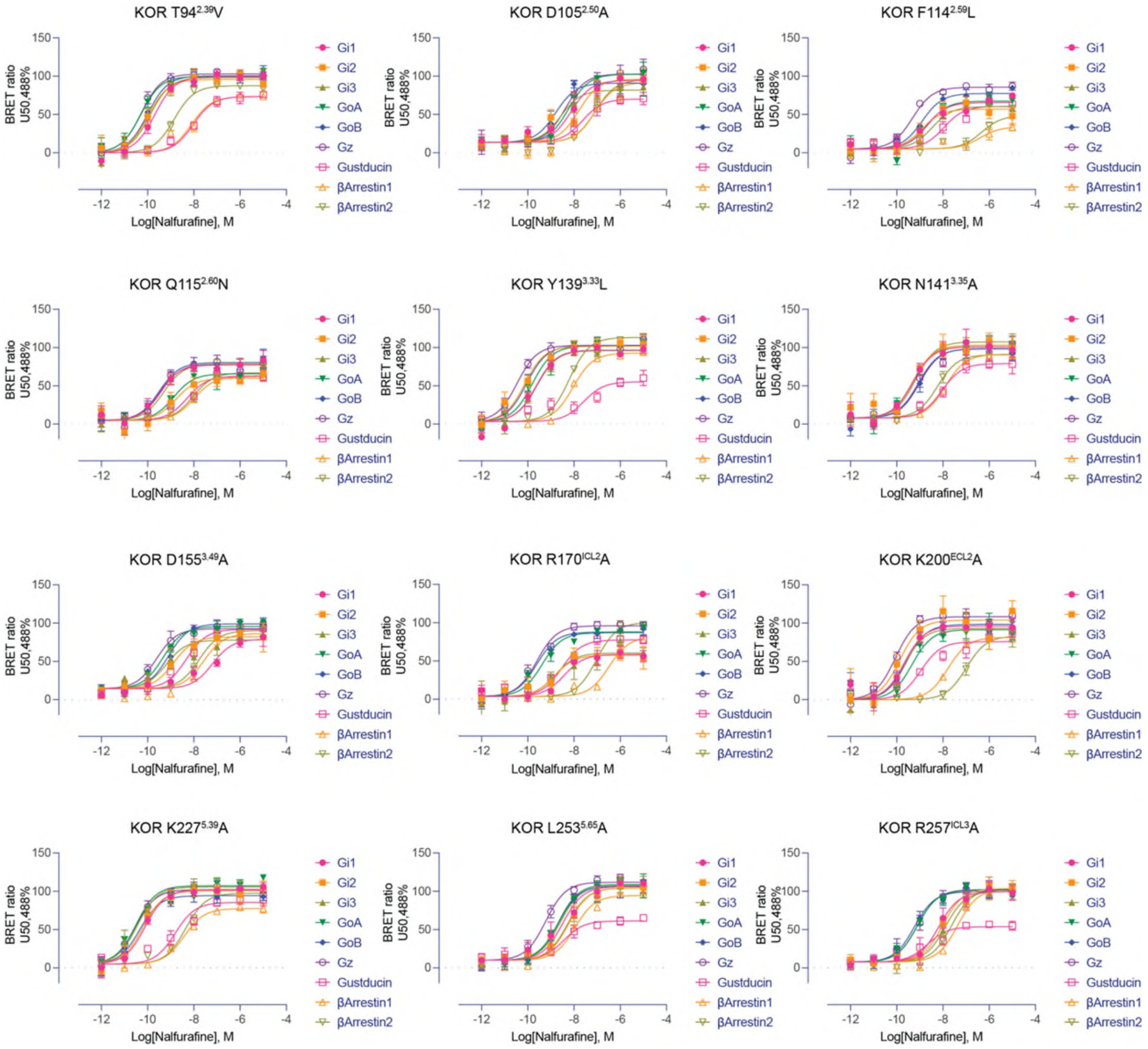

Supplemental Figure 8. Effects of indicated residues on the KOR-G protein subtype interactions (Con't). Data are expressed as the mean ± SEM of three independent experiments (n = 3 experiments each done in duplicate). Expression of KOR mutants were also quantitated by single-point radioligand binding assays. ^{3}H-Diprenorphine was used at 5 nM. Values in each plot were summarized in Supplemental Table 10-11.

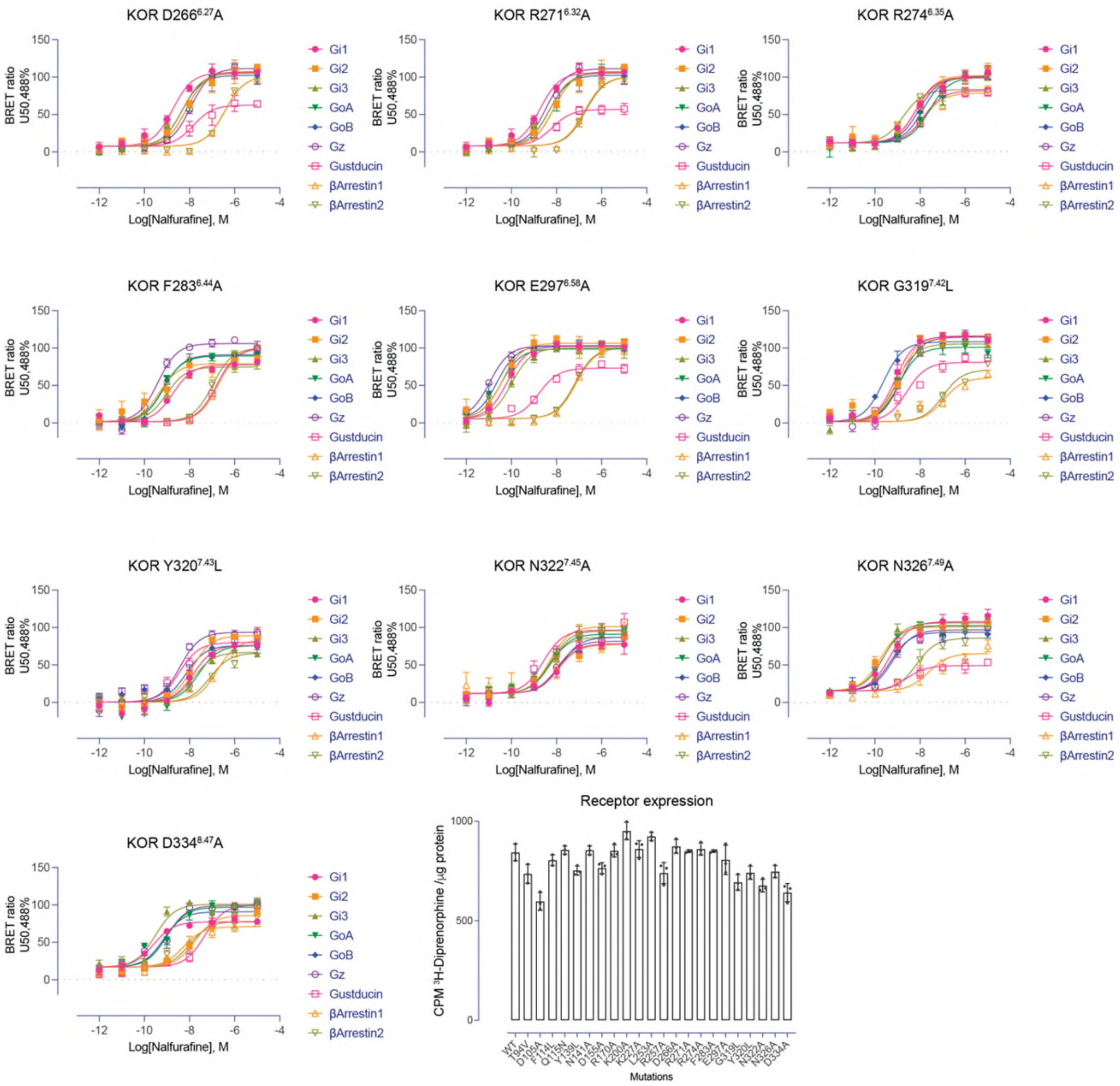

Supplemental Figure 9. Compatibility of the occluded state with arrestin and G protein coupling. Renderings of models of the KOR occluded state and arrestin or G protein complex modeled based on experimentally determined structures of (**a**) M2–β-arrestin 1 (PDB: 6U1N), (**b**) NTSR1–β-arrestin 1 (PDB: 6UP7), and (**c**) MOR–Gi1 (PDB: 6DDF). Models were generated by aligning an occluded state structure of KOR from a simulation frame to the receptor present in the indicated experimentally determined structure using PyMOL and then deleting the receptor from the experimentally determined complex structure.

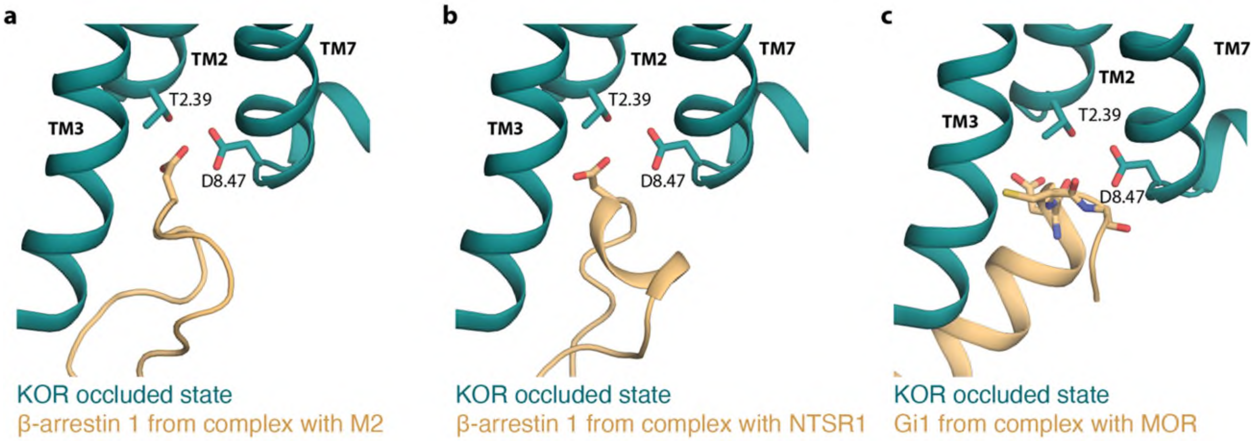

Supplemental Tables

Supplemental Table 1. Data Collection and Refinement Statistics.

**Structure**	**BRIL-KOR-Nalfurafine-Nb39**
**Data Collection**	APS, GMCA/CAT 23ID-B/D, 1.033 Å, 10-µm microfocus beam
Crystals	23
Resolution (Å)	38.38-3.30 (3.56-3.30)
Space group	$P22_12_1$
Complexes/ASU	2
Unit cell dimensions *a, b, c* (Å)	70.3 76.4 161.7
α, β, γ (°)	90.0 90.0 90.0
No. total reflections	40,881 (1,594)
No. unique reflections	10,443 (640)
Multiplicity	6.4 (4.4)
Completeness (%)	94.5 (88.7)
Mean I/σ(I)	5.2 (0.8)
R_{merge} (%)	18.2 (146.5)
$CC_{1/2}$ (%)	99.4 (49.6)
**Refinement Statistics**	
Resolution used in refinement (Å)	38.26-3.30 (3.21-3.10)
No. reflections used in refinement	12,421 (1,106)
No. reflections used for R-free	1,244 (107)
R-work (%)	27.5 (40.1)
R-free (%)	32.6 (46.7)
**Number of atoms**	3,393
KOR	2,136
Nb39	743
BRIL	479
Nalfurafine	35
**Overall B-factors (Å^2)**	122.7
KOR	108.1
Nb39	137.9
BRIL	165.6
Nalfurafine	101.7
**Model Statistics**	
RMSD Bond (Å)	0.011
RMSD Bond (°)	1.36
Ramachandran Favored (%)[a]	95.1
Ramachandran Allowed (%)[a]	4.9
Ramachandran Outliers (%)[a]	0.0
Rotamer outliers (%)[a]	0.0
Molprobity score[a]	2.13

Highest-resolution shell is shown in parentheses.

[a]As defined in MolProbity.

Supplemental Table 2 (related to Figure 1). Summary of potency and efficacy values reported in Figure 1. Potency (Log EC50, M) and efficacy (% reference receptor maximal response) are derived from simultaneous fitting of all biological replicates (n = 3 experiments each done in duplicate).

Receptor	pK_i ± SEM (radioligand binding), M
KOR	6.86 ± 0.12
DOR	9.37 ± 0.07
MOR	7.88 ± 0.07
NOP	N.A.

N.A. no activity

Receptor	pEC_{50} ± SEM (BRET-Gi1 protein activation), M
KOR	10.19 ± 0.08
DOR	7.13 ± 0.24
MOR	8.29 ± 0.18
NOP	N.A.

N.A. no activity

Supplemental Table 3 (Related to Supplemental Figure 1). Summary of potency and efficacy values reported in Supplemental Figure 1. Potency (Log EC50, M) and efficacy (% reference receptor maximal response) are derived from simultaneous fitting of all biological replicates (n = 3 experiments each done in duplicate).

Mutations	cAMP inhibition	
	pEC_{50} ± SEM, M	E_{max}% ± SEM
KOR WT	11.71 ± 0.07	100 ± 2
KOR S324$^{7.47}$C	11.09 ± 0.06	99 ± 2

Receptor	pK_i ± SEM (radioligand binding), M
KOR	8.63 ± 0.05
KOR + Gi1	9.54 ± 0.04
KOR + Nb39	10.22 ± 0.03

Nalfurafine	cAMP inhibition		Arrestin recruitment	
	pEC_{50} ± SEM, M	E_{max}% ± SEM	pEC_{50} ± SEM, M	E_{max}% ± SEM
KOR WT	11.65 ± 0.09	100 ± 3	9.95 ± 0.22	100 ± 5
KOR D138$^{3.32}$A	7.84 ± 0.10	102 ± 4	6.55 ± 0.11	102 ± 7

Supplemental Table 4 (Related to Supplemental Figure 2). Summary of potency and efficacy values reported in Supplemental Figure 2. Potency (Log EC50, M) and efficacy (% reference receptor maximal response) are derived from simultaneous fitting of all biological replicates (n = 3 experiments each done in duplicate).

Nalfurafine	cAMP inhibition	
	pEC_{50} ± SEM, M	E_{max}% ± SEM
KOR WT	11.71 ± 0.07	100 ± 2
KOR E209^{ECL2}A	11.84 ± 0.08	100 ± 2
KOR S211^{ECL2}A	11.64 ± 0.09	107 ± 2

Nalfurafine	cAMP inhibition		Arrestin recruitment	
	pEC_{50} ± SEM, M	E_{max}% ± SEM	pEC_{50} ± SEM, M	E_{max}% ± SEM
KOR WT	11.71 ± 0.06	100 ± 3	9.95 ± 0.22	100 ± 5
KOR G319$^{7.42}$L	9.12 ± 0.12	103 ± 4	7.43 ± 0.31	2.6 ± 1.2
KOR Y320$^{7.73}$L	9.99 ± 0.06	105 ± 2	7.11 ± 0.25	66 ± 7

Supplemental Table 5 (Related to Supplemental Figure 3). Summary of potency and efficacy values reported in Supplemental Figure 3. Potency (Log EC50, M) and efficacy (% reference receptor maximal response) are derived from simultaneous fitting of all biological replicates (n = 3 experiments each done in duplicate).

Nalfurafine	cAMP inhibition		Arrestin recruitment	
	pEC_{50} ± SEM, M	E_{max}% ± SEM	pEC_{50} ± SEM, M	E_{max}% ± SEM
KOR WT	11.32 ± 0.20	94 ± 2	9.85 ± 0.23	92 ± 5
KOR W287$^{6.48}$A	10.98 ± 0.24	98 ± 4	7.82 ± 0.18	85 ± 7

Nalfurafine	cAMP inhibition		Arrestin recruitment	
	pEC_{50} ± SEM, M	E_{max}% ± SEM	pEC_{50} ± SEM, M	E_{max}% ± SEM
KOR WT	11.57 ± 0.11	100 ± 3	9.95 ± 0.22	100 ± 5
KOR F114$^{2.59}$L	9.68 ± 0.14	97 ± 4	7.85 ± 0.60	17 ± 3
KOR W124^{ECL1}L	9.29 ± 0.14	98 ± 3	7.64 ± 0.78	26 ± 4
KOR V134$^{3.28}$A	10.47 ± 0.17	93 ± 3	6.62 ± 0.12	76 ± 6

Supplemental Table 6 (related to Figure 3). Summary of potency and efficacy values reported in Figure 3. Potency (Log EC50, M) and efficacy (% reference receptor maximal response) are derived from simultaneous fitting of all biological replicates (n = 3 experiments each done in duplicate).

Ligand	cAMP inhibition		Arrestin recruitment	
	pEC_{50} ± SEM, M	E_{max}% ± SEM	pEC_{50} ± SEM, M	E_{max}% ± SEM
Nalfurafine	11.52 ± 0.50	99 ± 2	9.55 ± 0.19	87 ± 4
U50,488	9.78 ± 0.12	99 ± 3	7.96 ± 0.51	100 ± 6
WMS-X600	9.98 ± 0.25	95 ± 3	9.33 ± 0.16	96 ± 5

Supplemental Table 7 (Related to Supplemental Figure 4). Summary of potency and efficacy values reported in Supplemental Figure 4. Potency (Log EC50, M) and efficacy (% reference receptor maximal response) are derived from simultaneous fitting of all biological replicates (n = 3 experiments each done in duplicate).

Ligand	BRET-Gi1		BRET-βArrestin2	
	pEC_{50} ± SEM, M	E_{max}% ± SEM	pEC_{50} ± SEM, M	E_{max}% ± SEM
Nalfurafine	10.31 ± 0.36	85 ± 7	8.59 ± 0.23	100 ± 4
U50,488	8.74 ± 0.26	98 ± 8	6.79 ± 0.13	71 ± 5
WMS-X600	8.85 ± 0.35	84 ± 8	7.51 ± 0.17	102 ± 6

Ligand	Mutations	cAMP inhibition		Arrestin recruitment	
		pEC_{50} ± SEM, M	E_{max}% ± SEM	pEC_{50} ± SEM, M	E_{max}% ± SEM
Nalfurafine	KOR WT	11.59 ± 0.07	100 ± 2	9.91 ± 0.21	100 ± 5
	KOR Q115$^{2.60}$N	10.41 ± 0.09	98 ± 3	8.47 ± 0.14	98 ± 3
WMS-X600	KOR WT	9.99 ± 0.14	96 ± 3	9.43 ± 0.16	110 ± 4
	KOR Q115$^{2.60}$N	11.16 ± 0.05	103 ± 1	10.11 ± 0.17	110 ± 6

Ligand	Mutations	cAMP inhibition		Arrestin recruitment	
		pEC_{50} ± SEM, M	E_{max}% ± SEM	pEC_{50} ± SEM, M	E_{max}% ± SEM
Nalfurafine	KOR WT	11.59 ± 0.07	100 ± 2	9.91 ± 0.21	88 ± 4
	KOR K227$^{5.39}$A	12.28 ± 0.09	100 ± 3	9.10 ± 0.14	89 ± 3

Supplemental Table 8 (related to Supplemental Figure 5). Summary of potency and efficacy values reported in Supplemental Figure 5. Potency (Log EC50, M) and efficacy (% reference receptor maximal response) are derived from simultaneous fitting of all biological replicates (n = 3 experiments each done in duplicate).

WMS-X600	cAMP inhibition		Arrestin recruitment	
	pEC_{50} ± SEM, M	E_{max}% ± SEM	pEC_{50} ± SEM, M	E_{max}% ± SEM
KOR WT	9.88 ± 0.12	92 ± 4	9.29 ± 0.14	92 ± 5
KOR W287$^{6.48}$A	6.96 ± 0.04	98 ± 5	6.52 ± 0.13	108 ± 7

U50,488	cAMP inhibition		Arrestin recruitment	
	pEC_{50} ± SEM, M	E_{max}% ± SEM	pEC_{50} ± SEM, M	E_{max}% ± SEM
KOR WT	9.48 ± 0.20	92 ± 4	7.36 ± 0.12	88± 6
KOR W287$^{6.48}$A	6.78 ± 0.31	98 ± 5	6.42 ± 0.23	96 ± 9

Supplemental Table 9 (related to Figure 5a). Summary of potency and efficacy values reported in Figure 3. Potency (Log EC50, M) and efficacy (% reference receptor maximal response) are derived from simultaneous fitting of all biological replicates (n = 3 experiments each done in duplicate).

Drug		Gαi1	Gαi2	Gαi3	GαoA	GαoB	GαZ	GαGust	β-Arrestin1	β-Arrestin2
U50,488	pEC_{50} ± SEM, M	8.97 ± 0.09	8.73 ± 0.22	8.71 ± 0.11	9.06 ± 0.16	9.12 ± 0.11	9.53 ± 0.21	7.22 ± 0.28	6.31 ± 0.07	6.86 ± 0.10
	Emax% ± SEM	99.98 ± 2.42	99.04 ± 5.70	99.96 ± 2.79	99.98 ± 4.30	100.0 ± 2.69	100.2 ± 4.65	99.69 ± 11.86	100.0 ± 3.67	99.99 ± 4.55
Nalfurafine	pEC_{50} ± SEM, M	10.26 ± 0.07	10.17 ± 0.11	10.17 ± 0.09	10.26 ± 0.14	10.63 ± 0.11	11.04 ± 0.13	8.44 ± 0.21	8.61 ± 0.20	8.96 ± 0.14
	Emax% ± SEM	101.1 ± 1.35	101.3 ± 2.76	100.1 ± 2.01	98.74 ± 2.47	99.79 ± 1.83	96.68 ± 2.20	65.57 ± 3.99	52.06 ± 2.89	87.39 ± 3.61
WMS-X600	pEC_{50} ± SEM, M	9.55 ± 0.08	9.19 ± 0.13	9.19 ± 0.07	9.29 ± 0.10	9.72 ± 0.08	10.01 ± 0.14	8.31 ± 0.24	7.28 ± 0.08	8.05 ± 0.10
	Emax% ± SEM	101.3 ± 2.02	99.35 ± 3.47	99.22 ± 1.94	99.81 ± 2.56	99.18 ± 1.93	97.70 ± 3.2	93.59 ± 7.13	93.8 ± 4.90	108.1 ± 4.70
6’-GNTI	pEC_{50} ± SEM, M	9.71 ± 0.08	9.70 ± 0.15	9.50 ± 0.13	9.71 ± 0.13	10.01 ± 0.07	10.13 ± 0.15	N.A.	N.A.	9.72 ± 0.32
	Emax% ± SEM	82.61 ± 1.62	73.62 ± 2.48	76.36 ± 2.49	79.00 ± 2.51	86.65 ± 1.66	87.90 ± 2.88	N.A.	N.A.	15.02 ± 1.56

N.A. no activity

Supplemental Table 10 (Potency related to Figure 5b). FC: Fold change compared to wild type.

Nalfurafine in KOR mutation	pEC_{50} ± SEM, M																	
	Gαi1		Gαi2		Gαi3		GαoA		GαoB		GαZ		GαGust		β-Arrestin1		β-Arrestin2	
		FC		FC		FC		FC		FC		FC		FC		FC		FC
KOR **WT**	10.26 ± 0.07	1	10.17 ± 0.11	1	10.17 ± 0.09	1	10.26 ± 0.14	1	10.63 ± 0.11	1	11.04 ± 0.13	1	8.44 ± 0.21	1	8.61 ± 0.20	1	8.96 ± 0.14	1
**T94$^{2.39}$V**	9.69 ± 0.12	4	9.99 ± 0.11	2	9.90 ± 0.12	2	10.30 ± 0.09	1	10.01 ± 0.11	4	10.28 ± 0.11	6	8.03 ± 0.18	3	8.12 ± 0.34	3	8.85 ± 0.18	1
**D105$^{2.50}$A**	8.12 ± 0.22	138	7.80 ± 0.21	234	8.57 ± 0.25	40	8.22 ± 0.16	110	8.79 ± 0.17	69	8.36 ± 0.19	479	7.63 ± 0.29	6	7.10 ± 0.30	32	7.06 ± 0.25	79
**F114$^{2.59}$L**	8.71 ± 0.23	35	8.91 ± 0.20	18	8.47 ± 0.22	50	8.66 ± 0.18	40	8.90 ± 0.17	54	9.31 ± 0.24	54	7.96 ± 0.25	3	6.39 ± 0.20	166	6.32 ± 0.23	437
**Q115$^{2.60}$N**	9.51 ± 0.19	6	8.79 ± 0.21	24	9.31 ± 0.15	7	8.88 ± 0.28	24	9.51 ± 0.19	13	9.45 ± 0.16	39	8.31 ± 0.32	1	7.99 ± 0.31	4	8.04 ± 0.21	8
**Y139$^{3.33}$L**	9.65 ± 0.23	4	10.15 ± 0.15	1	9.63 ± 0.14	3	9.80 ± 0.09	3	10.07 ± 0.13	4	10.48 ± 0.13	4	7.70 ± 0.35	5	8.06 ± 0.18	4	8.22 ± 0.16	5
**N141$^{3.35}$A**	9.30 ± 0.15	9	9.30 ± 0.14	7	9.19 ± 0.13	10	9.32 ± 0.15	9	9.01 ± 0.15	42	9.07 ± 0.16	93	8.05 ± 0.22	2	7.89 ± 0.23	5	8.23 ± 0.24	5
**D155$^{3.49}$A**	7.19 ± 0.32	1175	8.87 ± 0.20	20	9.19 ± 0.28	10	9.33 ± 0.18	9	9.10 ± 0.18	34	9.61 ± 0.17	27	8.32 ± 0.21	1	7.57 ± 0.23	11	7.77 ± 0.23	15
**R170^{ICL2}A**	8.44 ± 0.22	66	8.55 ± 0.25	42	8.52 ± 0.28	45	9.51 ± 0.16	6	9.74 ± 0.16	8	9.94 ± 0.14	13	8.47 ± 0.33	1	6.49 ± 0.28	132	7.02 ± 0.21	87
**K200^{ECL2}A**	9.68 ± 0.20	4	9.87 ± 0.18	2	9.66 ± 0.18	3	9.34 ± 0.20	8	9.59 ± 0.20	11	10.07 ± 0.18	9	8.90 ± 0.33	3	7.79 ± 0.25	7	6.99 ± 0.28	93
**K227$^{5.39}$A**	10.12 ± 0.12	1	10.22 ± 0.11	1	10.46 ± 0.16	2	10.52 ± 0.14	1	10.36 ± 0.14	2	10.54 ± 0.13	3	8.83 ± 0.19	2	8.42 ± 0.22	2	8.35 ± 0.19	4
**L253$^{5.65}$A**	8.48 ± 0.19	60	8.23 ± 0.16	87	8.27 ± 0.17	79	8.67 ± 0.15	39	8.67 ± 0.17	91	9.22 ± 0.13	66	8.44 ± 0.29	1	7.96 ± 0.23	4	8.60 ± 0.19	2
**R257^{ICL3}A**	8.18 ± 0.13	120	8.01 ± 0.13	145	7.90 ± 0.12	186	9.11 ± 0.12	14	9.23 ± 0.13	25	9.11 ± 0.13	85	8.68 ± 0.22	2	7.37 ± 0.17	17	7.62 ± 0.27	22
**D266$^{6.27}$A**	8.74 ± 0.13	33	8.13 ± 0.13	110	8.31 ± 0.16	72	8.14 ± 0.13	132	8.34 ± 0.16	195	7.94 ± 0.12	1259	7.84 ± 0.26	4	6.42 ± 0.21	155	6.44 ± 0.20	331
**R271$^{6.32}$A**	8.77 ± 0.13	33	8.16 ± 0.14	112	8.35 ± 0.17	74	8.16 ± 0.13	135	8.38 ± 0.15	195	8.49 ± 0.11	398	8.15 ± 0.36	2	6.75 ± 0.29	71	6.69 ± 0.18	178
**R274$^{6.35}$A**	8.18 ± 0.16	120	8.16 ± 0.16	102	8.18 ± 0.15	98	7.58 ± 0.17	479	7.87 ± 0.14	575	8.05 ± 0.15	977	7.84 ± 0.14	4	8.07 ± 0.21	3	8.73 ± 0.22	2
**F283$^{6.44}$A**	8.72 ± 0.14	35	9.34 ± 0.11	7	9.02 ± 0.16	14	9.16 ± 0.11	13	9.13 ± 0.11	32	9.40 ± 0.10	44	6.79 ± 0.11	45	6.72 ± 0.17	78	6.97 ± 0.16	98
**E297$^{6.58}$A**	10.20 ± 0.11	1	10.41 ± 0.11	2	9.98 ± 0.12	2	10.47 ± 0.14	2	10.64 ± 0.12	1	10.96 ± 0.11	1	8.78 ± 0.18	2	7.12 ± 0.17	31	7.18 ± 0.17	60
**G319$^{7.42}$L**	9.09 ± 0.14	15	8.94 ± 0.15	17	9.06 ± 0.17	13	8.95 ± 0.14	20	9.62 ± 0.18	10	8.83 ± 0.16	162	8.61 ± 0.23	1	6.95 ± 0.28	46	6.98 ± 0.24	95
**Y320$^{7.43}$L**	7.82 ± 0.18	275	7.90 ± 0.15	186	7.80 ± 0.20	234	7.60 ± 0.19	457	8.16 ± 0.22	295	8.43 ± 0.14	407	8.45 ± 0.28	1	6.78 ± 0.28	68	7.08 ± 0.25	76
**N322$^{7.45}$A**	7.93 ± 0.20	214	7.92 ± 0.24	178	8.26 ± 0.19	81	8.26 ± 0.17	100	7.82 ± 0.21	646	7.94 ± 0.20	1259	8.59 ± 0.19	1	8.27 ± 0.23	2	8.33 ± 0.25	4
**N326$^{7.49}$A**	9.32 ± 0.15	9	9.76 ± 0.16	3	9.61 ± 0.11	4	9.65 ± 0.13	4	9.31 ± 0.14	21	9.20 ± 0.13	69	8.58 ± 0.32	1	7.63 ± 0.37	10	8.16 ± 0.27	6
**D334$^{8.47}$A**	9.56 ± 0.20	5	7.74 ± 0.21	269	9.50 ± 0.12	5	9.00 ± 0.18	18	9.11 ± 0.24	33	8.99 ± 0.15	112	7.39 ± 0.15	11	8.23 ± 0.25	2	7.95 ± 0.33	10

Supplemental Table 11 (Efficacy related to Figure 5b) (Con't).

Nalfurafine in KOR mutation	E_{max}% ± SEM								
	Gαi1	Gαi2	Gαi3	GαoA	GαoB	GαZ	GαGust	β-Arrestin1	β-Arrestin2
KOR **WT**	100 ± 3.24	101 ± 2.92	99.93 ± 2.37	98.31 ± 3.11	99.55 ± 1.99	96.48 ± 2.47	65.57 ± 3.99	52.03 ± 2.94	87.39 ± 3.61
**T94$^{2.39}$V**	99.23 ± 3.20	95.8 ± 3.11	98.21 ± 3.14	99.67 ± 3.03	100.5 ± 2.62	102 ± 3.02	73.58 ± 4.22	73.23 ± 6.19	87.51 ± 5.03
**D105$^{2.50}$A**	94.34 ± 5.36	96.07 ± 5.72	81.72 ± 4.87	102.5 ± 5.10	91.01 ± 4.55	102.8 ± 5.07	70.15 ± 5.77	91.64 ± 9.45	95.38 ± 9.42
**F114$^{2.59}$L**	65.55 ± 3.91	56.89 ± 3.65	60.33 ± 3.97	67.41 ± 3.81	77.46 ± 3.74	85.56 ± 3.49	60.6 ± 4.59	33.89 ± 9.96	48.7 ± 10.22
**Q115$^{2.60}$N**	77.33 ± 3.76	59.98 ± 4.08	78.71 ± 3.80	65.77 ± 4.01	80.49 ± 3.77	78.65 ± 3.72	62.9 ± 4.80	62.25 ± 6.61	67.02 ± 6.68
**Y139$^{3.33}$L**	96.72 ± 3.66	101.7 ± 3.49	95.97 ± 3.69	101.9 ± 3.62	102.4 ± 3.50	102.8 ± 3.29	55.31 ± 6.35	92.81 ± 6.53	113.3 ± 6.40
**N141$^{3.35}$A**	100.9 ± 3.98	103.1 ± 4.04	107.4 ± 4.10	101.1 ± 4.03	98.71 ± 4.17	98.3 ± 4.18	79.1 ± 5.00	91.26 ± 7.09	90.62 ± 6.65
**D155$^{3.49}$A**	79.07 ± 6.38	82.03 ± 4.00	77.84 ± 4.04	95.68 ± 3.84	99.05 ± 3.97	92.73 ± 3.62	91.82 ± 4.58	86.45 ± 6.93	90.18 ± 6.73
**R170^{ICL2}A**	58.67 ± 4.37	57.62 ± 4.12	60.3 ± 4.30	86.94 ± 4.00	87.79 ± 3.81	95.95 ± 3.82	77.48 ± 4.54	81.8 ± 10.44	100.4 ± 8.6
**K200^{ECL2}A**	96.13 ± 5.54	103.7 ± 5.37	93.07 ± 5.46	91.42 ± 5.72	98.32 ± 5.59	108.3 ± 5.30	75.72 ± 6.34	81.34 ± 10.47	83.17 ± 12.86
**K227$^{5.39}$A**	102.2 ± 3.22	100.6 ± 3.16	105.6 ± 3.10	107.3 ± 3.20	94.35 ± 3.17	102.2 ± 3.12	85.50 ±4.01	77.46 ± 5.85	97.55 ± 5.96
**L253$^{5.65}$A**	106.1 ± 4.2	104.6 ± 4.20	104.4 ± 4.30	109 ± 3.80	106.8 ± 3.80	111.9 ± 3.60	61.36 ± 4.02	94.50 ± 6.19	107.40 ± 5.40
**R257^{ICL3}A**	99.49 ± 3.87	103.5± 4.04	100 ± 4.08	102.2 ± 3.32	99.60 ± 3.31	101.6 ± 3.39	53.79 ± 3.91	103.2 ± 6.52	103.2 ± 6.02
**D266$^{6.27}$A**	106 ± 3.80	107.3 ± 4.20	105.2 ± 4.30	107.6 ± 4.30	102.4 ± 4.18	111.7 ± 4.30	62.59 ± 4.53	101.5 ± 9.54	101.3 ± 9.39
**R271$^{6.32}$A**	105 ± 3.70	106.4 ± 4.20	103.3 ± 4.20	106.7 ± 4.20	101.5 ± 4.12	110.2 ± 4.00	56.9 ± 4.24	100.9 ± 8.05	101 ± 8.20
**R274$^{6.35}$A**	100.1 ± 4.05	102 ±4.03	98.85 ± 3.93	99.55 ± 4.64	101.2 ± 4.19	99.13 ±4.01	81.96 ± 4.08	78.68 ± 6.01	83.35 ± 4.94
**F283$^{6.44}$A**	77.61 ± 3.32	79.03 ± 3.30	74.75 ± 3.23	89.50 ± 3.09	91.02 ± 3.10	106 ± 3.00	100.2 ± 5.19	100.2 ± 7.42	100.2 ± 6.90
**E297$^{6.58}$A**	102.4 ± 2.80	106.5 ± 2.80	100.2 ± 2.83	98.88 ± 2.80	103 ± 2.70	103.1 ± 2.60	73.14 ± 3.44	100.6 ± 6.72	100.7 ± 6.68
**G319$^{7.42}$L**	115.7 ± 5.00	116.3 ± 5.10	105.5 ± 5.10	101.1 ± 5.05	108.3 ± 4.70	114.8 ± 5.20	80.92 ± 5.98	60.42 ± 13.31	70.99 ± 11.83
**Y320$^{7.43}$L**	76.32 ± 5.02	89.34 ± 4.92	66.88 ± 5.05	75.75 ± 5.28	75.99 ± 4.86	93.72 ± 4.43	80.02 ± 4.94	83.58 ± 9.96	65.94 ± 9.47
**N322$^{7.45}$A**	78.39 ± 4.48	77.25 ± 4.67	95.77 ± 4.40	91.34 ± 4.28	87.11 ± 4.80	81.81 ± 4.55	96.59 ± 4.21	101.3 ± 6.13	87.09 ± 5.91
**N326$^{7.49}$A**	107.8 ± 3.40	101 ± 3.19	106.2 ± 3.20	102.6 ± 3.19	93.63 ± 3.31	96.86 ± 3.36	49.16 ± 3.77	65.16 ± 6.76	85.75 ± 5.71
**D334$^{8.47}$A**	77.45 ± 3.23	86.08 ± 4.41	101 ± 3.16	96.92 ± 3.62	91.10 ± 3.75	99.23 ± 3.52	100.1 ± 4.86	71.06 ± 5.64	78.02 ± 6.04
